# Evaluating the Role of Anharmonic Vibrations in Zeolite β Materials

**DOI:** 10.1021/acs.jpcc.3c02863

**Published:** 2023-08-07

**Authors:** Owain T. Beynon, Alun Owens, Christian Carbogno, Andrew J. Logsdail

**Affiliations:** † Cardiff Catalysis Institute, Cardiff University, Park Place, Cardiff CF10 3AT, Wales, U.K.; ‡ The NOMAD Laboratory at the FHI of the Max-Planck-Gesellschaft and IRIS-Adlershof of the Humboldt-Universität zu Berlin, Faradayweg 4-6, 14195 Berlin, Germany

## Abstract

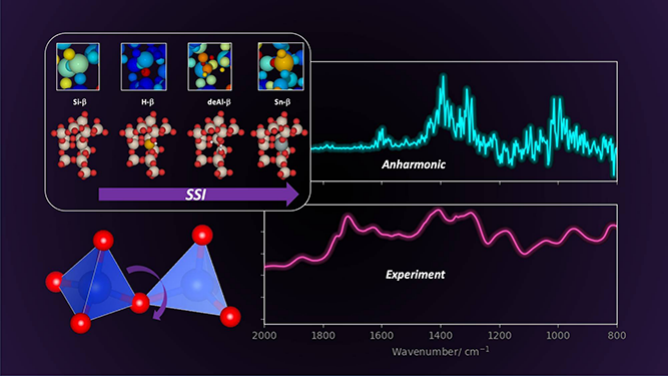

The characterization of zeolitic materials is often facilitated by spectroscopic analysis of vibrations, which informs about the bonding character of the substrate and any adsorbents. Computational simulations aid the interpretation of the spectra but often ignore anharmonic effects that can affect the spectral characteristics significantly. Here, the impact of anharmonicity is demonstrated with a combination of dynamical and static simulations applied to the structures formed during the synthesis of Sn-BEA *via* solid-state incorporation (SSI): the initial siliceous BEA (Si-β), aluminosilicate BEA (H-β), dealuminated BEA (deAl-β), and Sn-BEA (Sn-β). Heteroatom and defect-containing BEA are shown to have strong anharmonic vibrational contributions, with atomic and elemental resolution highlighting particularly the prevalence for H atoms (H-β, deAl-β) as well as localization to heteroatoms at defect sites. We simulate the vibrational spectra of BEA accounting for anharmonic contributions and observe an improved agreement with experimental data compared to harmonic methods, particularly at wavenumbers below 1500 cm^–1^. The results demonstrate the importance of incorporating anharmonic effects in simulations of vibrational spectra, with consequences toward future characterization and application of zeolitic materials.

## 1 Introduction

**Figure 1:**
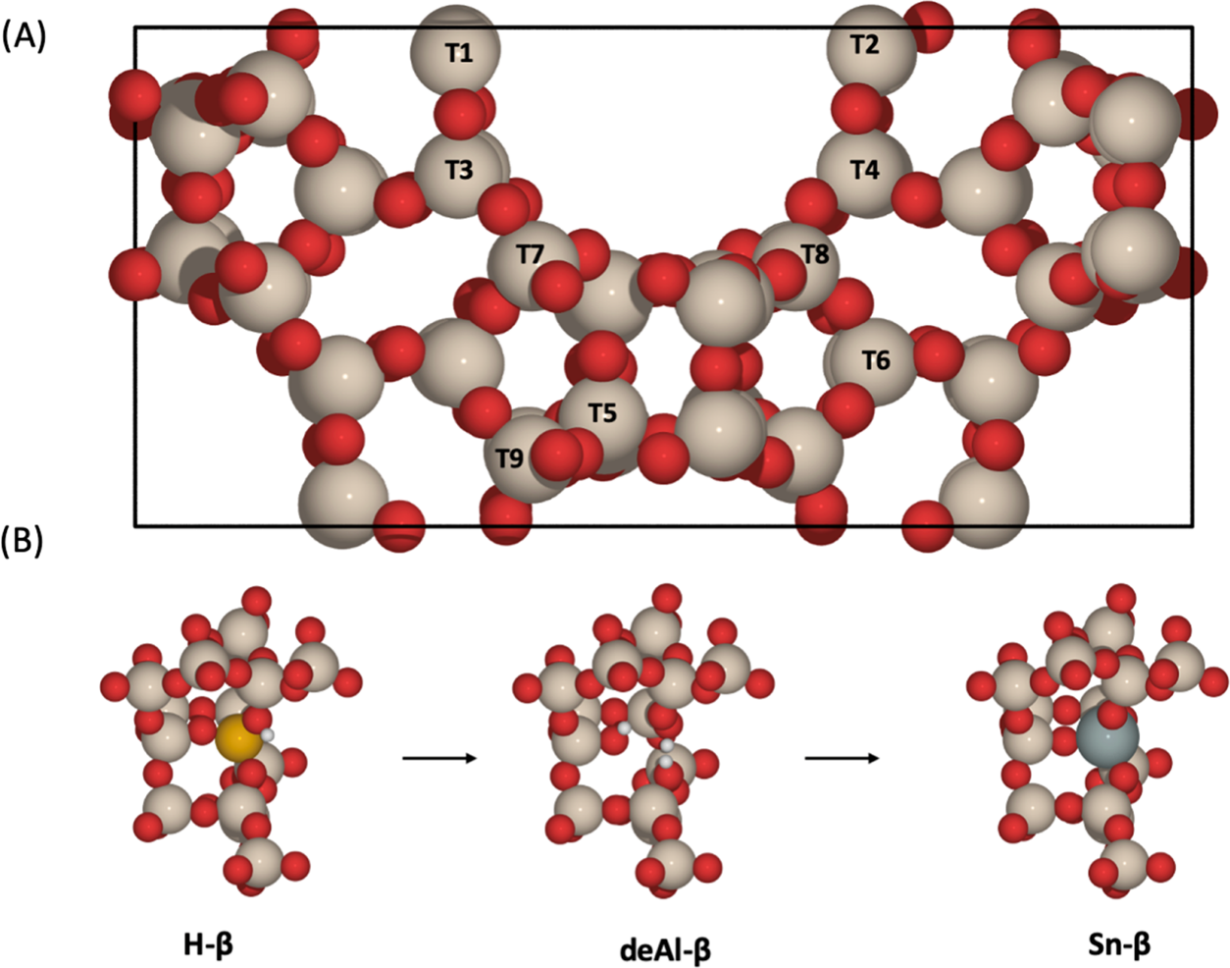
(A): Unit cell of BEA with the nine distinct crystallographic tetrahedral (T-) sites highlighted. (B): Change of the T2 active site of BEA during solid-state incorporation (SSI). The red, beige, white, black, orange, and gray atoms represent O, Si, H, C, Al, and Sn, respectively.

Zeolite catalysts can catalyze several important reactions in green and sustainable chemistry. Heteroatom-doped zeolite frameworks, such as zeolite β (BEA), display potent catalytic ability, especially when Lewis acid elements such as Ti, Sn, or Ge are incorporated in the BEA framework (Figure 1). Of these, zeolite Sn-BEA (Sn-β) has emerged as a state-of-the-art catalyst for reactions such as the Baeyer–Villiger oxidation (BVO), the Meerwein–Ponndorf–Verley (MPV) reduction, and the isomerization of sugars, which are all considered important reactions for upgrading biomass.^1−10^

Recently, the demonstration of solid-state incorporation (SSI) for synthesizing Sn-β has addressed limitations in wet synthesis that hinder scale-up, such as high temperatures and pressures and strong acids.^11−14^ SSI is a “top-down” synthetic approach where readily available zeolite BEA (H-β) undergoes dealumination with a strong acid, creating vacancies in the framework (silanol nests) at the sites previously occupied by Al. The dealuminated framework (deAl-β) then undergoes mechanochemical grinding with a Sn precursor (Sn(II) acetate), which induces interaction with the framework, and subsequent heating and cooling steps further incorporate Sn into the BEA framework to form the final catalyst (Sn-β, Figure 1B). A combination of *in situ* and *ab initio* spectroscopic studies have been vital in characterizing the mechanism and intermediates during SSI, with a comparison between infrared spectra and vibrational simulation highlighting key stages such as a change in Sn(II) acetate coordination upon grinding, from bidentate to monodentate, and the formation of acetic acid.^14^

When performing *ab initio* vibrational simulations of materials, the calculations often rely on a harmonic approximation, where the potential energy surface (PES), *V*(**R**), around the equilibrium positions, **R**_**0**_, is approximated *via* a second-order Taylor expansion, *i.e.*, by a parabolic potential
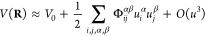
1Here, *V*_0_ = *V*(**R**_**0**_) is the potential energy in equilibrium and ***u***_i_^α^ = **R**_i_^α^ – **R**_0,i_^α^ are small displacements from equilibrium. Greek letters represent Cartesian directions (*i.e.*, α = *x, y, z*)*, i* and *j* are atoms within the lattice, and Φ_*i*,*j*_^α,β^ represents the harmonic force constants, *i.e.*, the Hessian of the PES evaluated at **R**_**0**_. The mass-scaled Fourier transform of Φ yields the dynamical matrix **D**(**q**); its eigenvalues and eigenvectors determine the harmonic vibrational modes.

The harmonic approximation is computationally advantageous and formally appealing since the nuclear equations of motion can be solved analytically. When applied with semi-local density functional theory (DFT), the harmonic approximation can yield surprisingly good quantitative agreement with experimental vibrational spectra; however, the agreement with experiment has been traced back to a benign error cancellation between the red shift caused by the too-shallow PES, from semi-local DFT, and the blue shift typically observed due to the deficiencies of the harmonic approximation.^15,16^ In spite of the experimental agreement, the qualitative validity of the harmonic approximation is therefore severely limited.^17^ For instance, modeling and understanding material properties such as heat transport, phase transitions, and thermal lattice expansion typically requires accounting for so-called anharmonic effects, beyond the harmonic approximation. Similarly, the harmonic approximation only holds for small displacements from equilibrium, *e.g*., in the low-temperature limit. Under more catalytically relevant thermodynamic conditions, the assumptions do not hold true for many materials,^18^ especially for materials featuring soft bonds and hence quite mobile atoms, such as zeolites.^16^ In this case, accounting for anharmonic effects is essential to reproduce vibrational modes and adsorption free energies.^20−23^ Furthermore, exothermic/endothermic catalytic processes can lead to a respective localized increase/decrease of temperature in such materials and, accordingly, to local changes in anharmonicity that are desirable to quantify. Developing knowledge of the role of anharmonic effects in materials such as zeolites can lead to a better understanding of how energy is generated and dissipated and how this affects catalytic properties.

The role of anharmonic effects is typically discussed in terms of macroscopic observables, for instance, these effects have previously been investigated by inspecting adsorption and diffusion of molecules in zeolites in consideration of thermodynamics.^19,21−24^ Additionally, experimental studies have quantified the significance of anharmonic effects in zeolite systems through observed changes in infrared (IR) signals, which is indicative of anharmonicity, though it can be challenging to interpret.^25,26^ In the work herein, we aim to computationally quantify the role of anharmonicity at a microscopic level by building on a recently proposed anharmonicity metric.^18^ The method improves connectivity between experimental and computational methods and facilitates a better understanding of mechanistic steps in chemical processes, which we demonstrate for the intermediates in the synthesis of Sn-β.

## 2 Computational Methods

### 2.1 Quantification of Anharmonicity

As previously mentioned, we seek to better our understanding of SSI through quantifying anharmonic behavior in zeolite β materials. In this approach, anharmonic effects, *V^A^*(**R**), are quantified at any given atomic configuration by measuring the difference between the correct and harmonic PES, *V*^(2)^ (**R**), *i.e.*, *via*
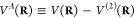
2

**Figure 2:**
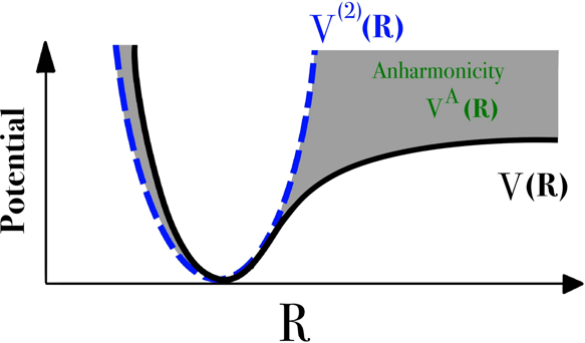
Potential energy surface (PES), *V*(**R**), harmonic approximation, *V*^(2)^(**R**), and the difference, highlighted in gray, defined as anharmonicity, *V*^*A*^(**R**).

The approach, as represented pictorially in Figure 2, sheds light on the role of anharmonic effects within the zeolite framework itself and its consequences for catalytic activity, allowing anharmonic effects to be quantified within siliceous BEA and the various framework species formed during the SSI process to synthesize Sn-β. In doing so, anharmonicity is measured relative to the framework of BEA, and changes in anharmonicity throughout SSI are tracked on the catalytic active site. By quantifying the anharmonic nature of BEA and framework products formed in SSI, we move toward the evaluation of vibrational models and methods in simulating zeolites, especially for the comparison with experimental vibrational spectra.

To evaluate anharmonic contributions in zeolites in terms of the anharmonicity metric, σ proposed by Knoop et al.,^18^ the following steps are performed: First, we obtain harmonic force constants Φ, see eq 1, in the low-temperature limit. Second, we sample the phase space explored at 300 K to quantify the actual harmonic and anharmonic forces experienced during the dynamics. Finally, we evaluate the anharmonicity metric
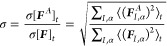
3where ***F*** and ***F***^*A*^ = ***F*** - ***F***^ha^ are the total and anharmonic force component α acting on atom *I* at time *t.* As this equation highlights, σ quantifies the difference between actual and harmonic forces
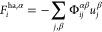
4acting on the system in thermodynamic equilibrium, with the difference normalized with respect to the average strength of the forces in thermodynamic equilibrium. Equation 3 can be considered equivalent to the root-mean-squared error (RMSE) of the harmonic model in relation to the standard deviation of the force distribution. Qualitatively, the anharmonicity metric, σ, captures how much anharmonic effects contribute to the total interactions in thermodynamic equilibrium; very harmonic materials may have σ < 0.1, where anharmonic contributions are on average less than 10% across all forces, while more anharmonic systems will have σ > 0.3, which indicates that the interactions are on average above 30% anharmonic. In principle, such an assessment of anharmonic effects could be performed by just comparing energies in line with eq 2; however, it is advantageous to use forces as in eq 3, since this allows more microscopic insights in terms of single atoms and phonon modes, as discussed below.

**Figure 3:**
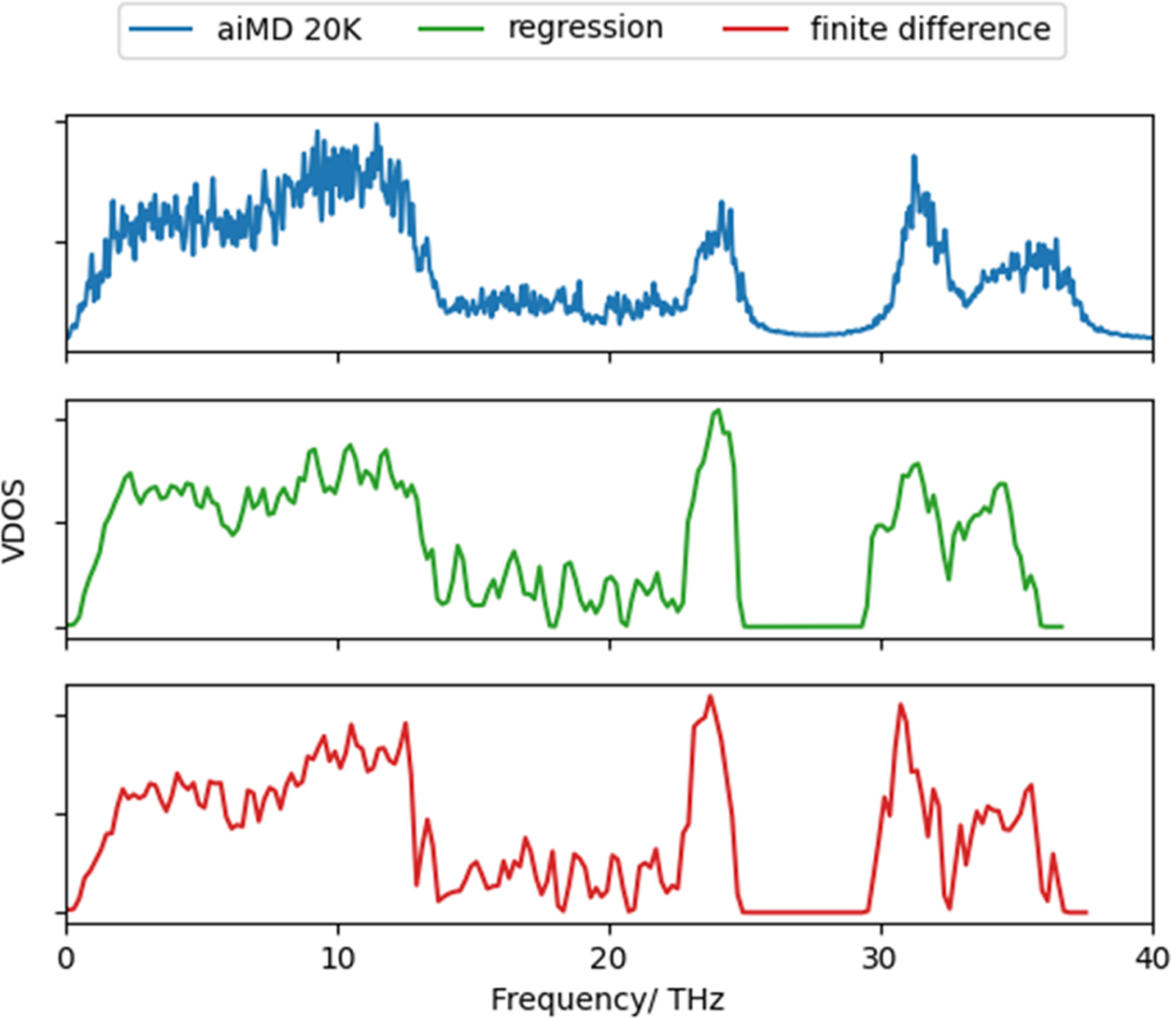
Vibrational density of states for Si-β, as calculated: from aiMD simulations at 20 K (blue line) *via* the velocity autocorrelation function; using regression methods (*hiPhive*, green line) *via* Gaussian smearing; using the finite-difference method with Γ-point sampling (phonopy, red line) *via* Gaussian smearing.

To evaluate eq 3 in practice, the approach described in ref (15) is adapted to be able to treat the strongly anharmonic zeolites. First, the harmonic force constants are extracted *via* regression^27^ using *hiPhive*(28) from *ab initio* MD (aiMD) simulations at low temperatures (20 K), rather than from finite-difference phonon calculations (Supporting Information, Figure S1). In this regression approach, eq 4 is numerically inverted to determine the force constants Φ that best reproduce the forces ***F***_*i*_(*t*) and displacements ***u***_*i*_(*t*) observed in the aiMD. The approach is demonstrated as essentially equivalent to standard phonon approaches, and for zeolites with greater anharmonicity (Figure 3), the use of this regression technique enables a consistent assessment of low-temperature force constants for all investigated systems. This approach is preferable for these materials since very “soft” lattice degrees of freedom and “hard” intramolecular degrees of freedom exhibit very different anharmonicity, resulting in a pronounced dependence of the obtained force constants on the chosen displacement in standard finite-difference phonon calculations. In these cases, the regression approach used herein provides a more balanced description of both soft and hard degrees of freedom.

**Figure 4:**
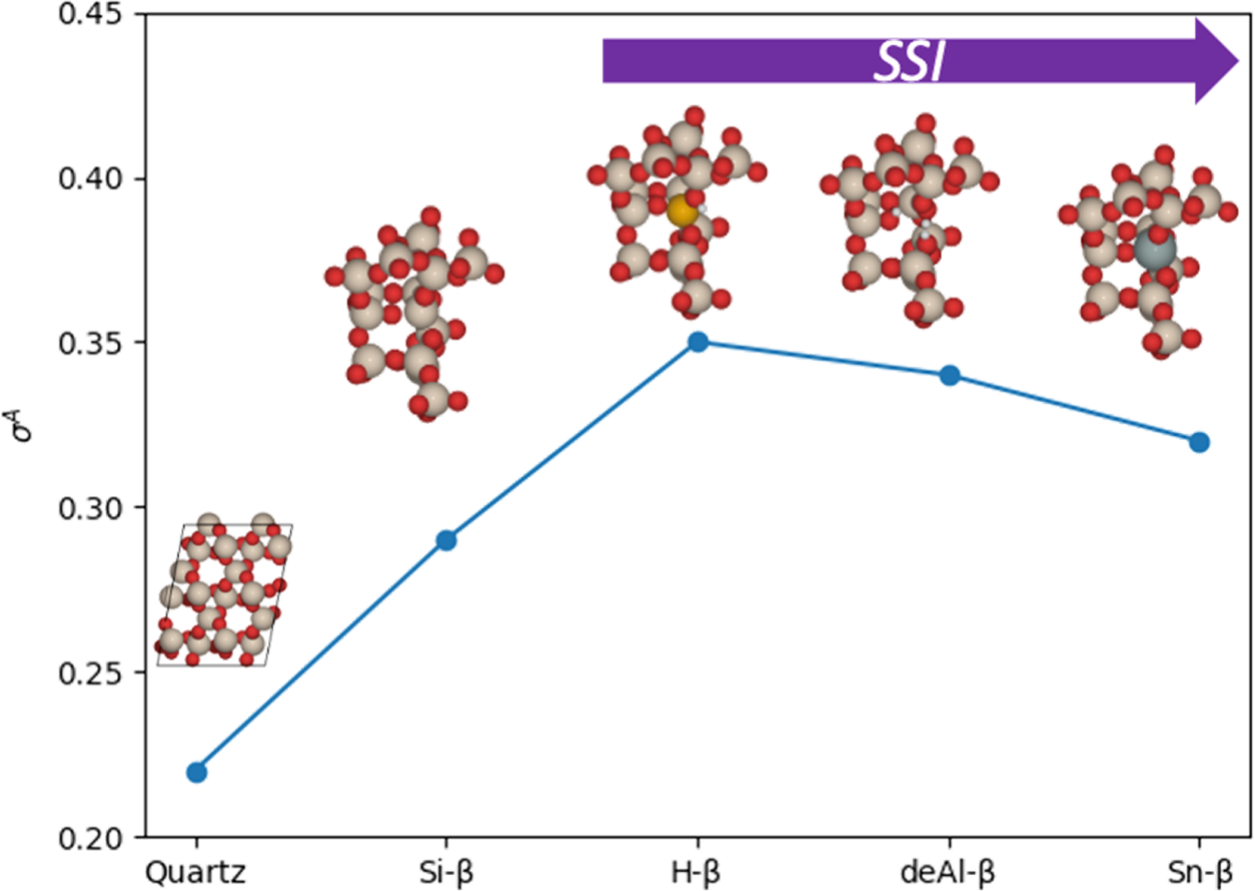
Overall anharmonicity score (σ^A^) for α-quartz and BEA materials. For all materials, harmonic force constants were obtained through regression methods, and the PES was evaluated using harmonic sampling at 300 K. A supercell of α-quartz (108 atoms) is shown, while only atoms to the fifth nearest-neighbor from the T2 site are depicted for the BEA systems. The red, beige, white, black, orange, and gray atoms represent O, Si, H, C, Al, and Sn, respectively.

Once low-temperature force constants have been obtained, an assessment of the anharmonicity requires sampling the fully anharmonic PES and computation of the associated forces. The corresponding harmonic forces, eq 4, are obtained from evaluating the displacement along the MD trajectory *via**u***_**i**_(*t*) = **R**_*i*_(*t*)–**R**_0,*i*_, so that the anharmonic contribution to the forces ***F***_i_^**A**^(*t*) = ***F**_i_*(*t*) – ***F***_i_^ha^(*t*), required for the evaluation of σ in eq 3, are also readily obtained. By thermodynamically averaging over the geometric configurations explored, accurate values of σ can be obtained. In principle, the most accurate approach for thermodynamically sampling the fully anharmonic PES is aiMD, see Figures S2 and S3 in the SI for aiMD at 300 K. However, as discussed below, zeolitic systems formally exhibit a very strong anharmonicity at 300 K (SI, Figure S4) owing to the strongly anharmonic lattice distortions present. These distortions are associated with the porosity of the material and the resulting softness of the lattice, as indicated by the low-frequency acoustic modes exhibiting the highest anharmonicity, *c.f.*Figure 4 and its discussion. These large acoustic distortions alter the overall lattice and, in turn, also affect the evaluation of the anharmonicity for all other degrees of freedom. To qualitatively understand this behavior and to ensure that local changes in anharmonicity are not overshadowed by global distortions of the soft lattice, we use the so-called harmonic sampling for phase space exploration.

In the harmonic sampling, the nuclear dynamics are sampled only using the harmonic approximation, rather than the fully anharmonic potential. The approach means that the thermodynamic average in eq 3 is performed by exploring the phase space given by the harmonic approximation in a Monte Carlo fashion.^29,30^ For our evaluation of the phonon modes of BEA, 50 samples were generated, which we find to be converged (SI, Figure S5). For the resulting configurations, ***F***_i_*^**A**^*(*t*) is evaluated *via* DFT so that insights on the acting anharmonic forces can be obtained (SI, Figure S6). Although the harmonic sampling approach is not as systematic as aiMD, it is advantageous for zeolites since the phonon picture is inherently retained. Accordingly, the strongly anharmonic acoustic modes mentioned are effectively constrained, which facilitates the disentanglement of the contributions affecting the other, more local phonon modes, and hence allows us to qualitatively inspect which phonon modes are most affected by anharmonicity, which we discuss below. However, in an unconstrained treatment of low-frequency vibrational modes through aiMD, greater anharmonic contributions are obtained from acoustic phonons, and thus, in the method described above, the anharmonicity values obtained serve as a lower estimate.

### 2.2 Simulation Details

DFT calculations were performed with the Fritz Haber Institute Ab Initio Molecular Simulation (FHI-aims) software package,^31^ which is an all-electron, full potential code. The general gradient approximation (GGA) exchange–correlation functional (XC) of Perdew–Burke–Ernzerhof, reparametrized for solids (PBEsol), was used,^32^ with dispersion interactions accounted for using the Tkatchenko–Scheffler method.^33^ Calculations were performed using a “light” basis set of the 2010 release, with self-consistent field (SCF) cycle convergence reached when the sum of eigenvalues and change in charge density were below 10^-6^ eV and 10^–6^ e/a_0_^3^, respectively. Calculations were also performed spin-restricted and using the atomic zeroth order regular approximation (ZORA) for relativistic treatments.^34^

The BEA unit cell, which contains 9 symmetry inequivalent tetrahedral (T) sites, was optimized from the structure first characterized by Newsam (*a, b* = 12.643 Å, *c* = 26.182 Å, 192 atoms),^35^ obtained from the database of International Zeolite Association (IZA). The α-quartz unit cell was obtained from the materials project database.^36^ Models were built and manipulated using the Atomic Simulation Environment (ASE) and FHI-vibes Python packages.^37,38^ Electronic structure calculations were performed with a converged Monkhorst–Pack ***k***-point sampling grid of 2 × 2 × 2 for BEA and 12 × 12 × 10 for α-quartz. Full unit cell optimizations were performed on all structures using the Broyden–Fletcher–Goldfarb–Shanno (BFGS) algorithm, with convergence reached when the forces on all atoms are less than a strict criterion of 0.001 eV Å^–1^,^39−42^ as variation in local minima would affect the definition of the harmonic approximation. For the BEA systems studied, geometry optimization was performed on Si-β, deAl-β (vicinal silanol groups), Sn(IV)-β (closed site), and H-β (12-membered ring with H oriented toward the pore); these structures can be found on the NOMAD repository (DOI: 10.17172/NOMAD/2023.06.30-1).

Anharmonic contributions were calculated using FHI-vibes, where anharmonicity was defined according to eq 2, and a metric for anharmonicity, σ, was defined according to eq 3. *Ab initio* molecular dynamics (aiMD) simulations were performed with FHI-vibes, FHI-aims, and ASE, using Langevin dynamics. Harmonic force constants from aiMD simulations were obtained by using the *hiPhive* Python package.^28^ Spectroscopic properties were obtained *via* the vibrational density of states (VDOS) using a Fourier transform of the velocity autocorrelation function
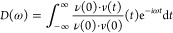
5

where *D*(ω) is the VDOS at frequency ω and ν is the velocity at time *t*.

The entropy of the reaction, Δ*S*, was calculated for the harmonic approximation and for anharmonicity at 300 K *via*
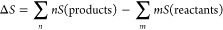
6where *n* and *m* are the stoichiometric coefficients for the reaction. Entropy values were obtained through normal mode analysis of aiMD trajectories at 20 K (harmonic) and 300 K (anharmonic) using the Phonopy package,^43^ where *S* was calculated using eq 11 in ref (43)

## 3 Results and Discussion

### 3.1 Total Anharmonicity

Our method has been applied to the BEA framework species present when forming Sn-BEA *via* SSI. The overall anharmonicity score (σ^A^) for siliceous BEA (Si-β) is 0.29, meaning that anharmonic effects contribute 29% of the total forces. Furthermore, σ^A^ are slightly greater for BEA frameworks produced during SSI, where the aluminosilicate (H-β), dealuminated (deAl-β), and Sn-containing (Sn-β) frameworks have anharmonicity scores of 0.35, 0.34, and 0.32, respectively (Figure 4); these frameworks are noted as descending in value of σ^A^. The greater anharmonic contributions for heteroatom and defect-containing BEA materials suggest that local effects, such as addition or removal of atoms, influence the overall anharmonic nature of the material. Moreover, it is noteworthy that all BEA materials have greater anharmonic contributions than dense silicate systems such as quartz, which has a σ^A^ value of 0.22, suggesting that the porous nature of the BEA framework is a key factor for the greater level of anharmonicity.

### 3.2 Vibrational Mode-Resolved Anharmonicity

**Figure 5:**
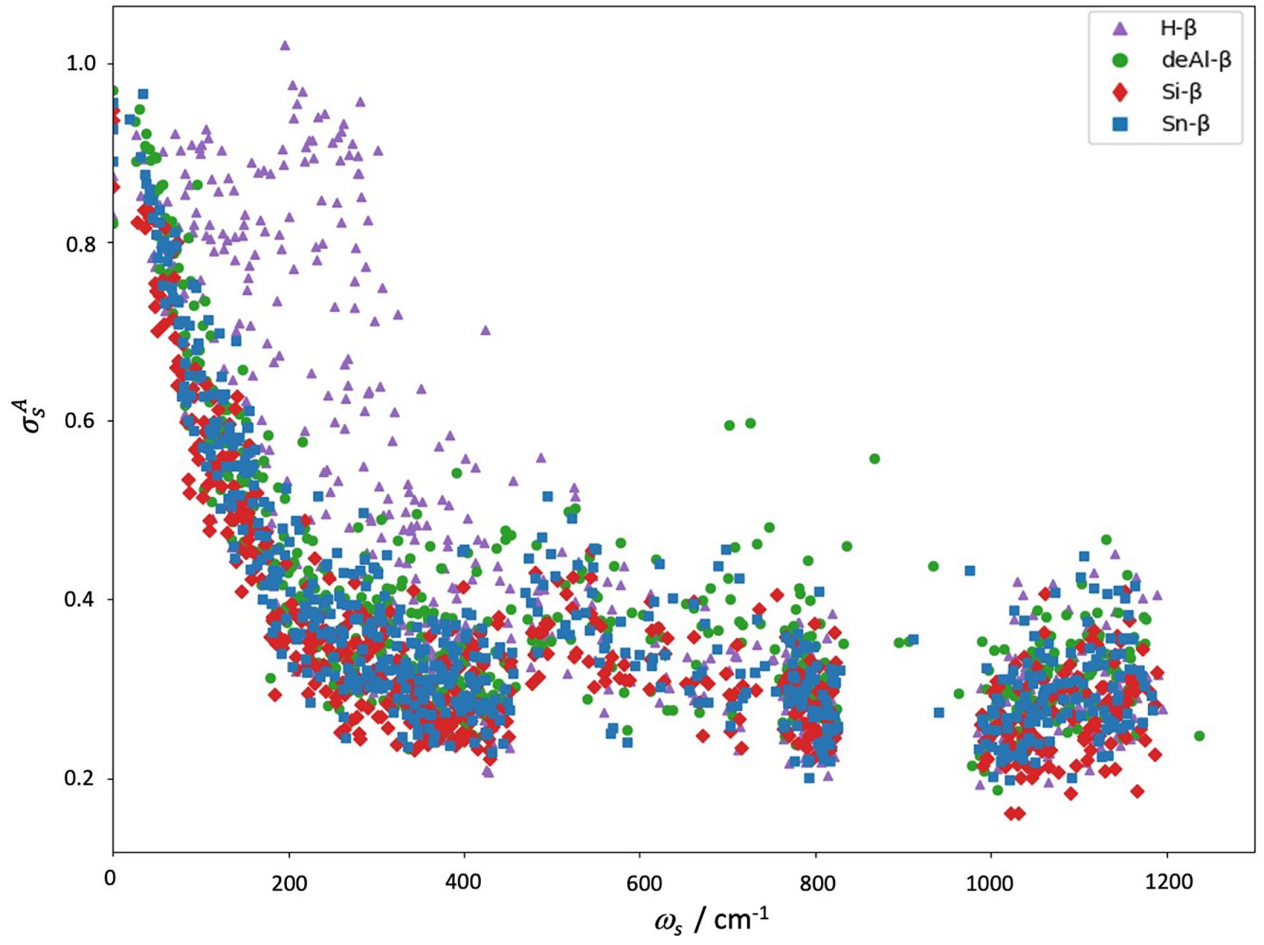
Mode-resolved anharmonicity, σ_s_^A^, for Sn-β (blue squares*)*, Si-β (red diamonds), deAl-β (green circle), and H-β (purple triangles). For all BEA materials, harmonic force constants were obtained through regression methods, and the PES was evaluated using harmonic sampling at 300 K.

In addition to the overall anharmonicity score (σ^A^), the anharmonicity per vibrational mode, σ_s_^A^, can be obtained from the mode-resolved force, ***F***_s_. Plotting σ_s_^A^ against the mode frequency (ω_s_) for Si-β, H-β, deAl-β, and Sn-β (Figure 5) shows that σ_s_^A^ changes significantly across the vibrational spectrum for all BEA systems. For Si-, Sn-, and deAl-β, frequency modes under 200 cm^–1^ are dominated by anharmonic contributions, with all values above 0.4, and for vibrational modes in the range of 1–10 cm^–1^, σ_s_^A^ is ∼1, which indicates the modes are entirely anharmonic. These soft, very anharmonic modes are associated with large global lattice distortions arising from the porosity of the material that cannot be accurately described in a harmonic model. Consequently, these modes would dominate the anharmonicity score when averaging over all modes. In our case, this is undesirable since we are not interested in studying the well-known softness of the zeolite lattice. Rather, we aim at understanding more local changes in the anharmonicity associated with variations of the chemical environment. In this more local vibrational regime, the large anharmonic contributions rapidly reduce and stabilize for ω_s_ > 200 cm^–1^, with an average σ_s_^A^ value of 0.29, 0.33, and 0.39, for Si-, Sn-, and deAl-β, respectively.

For H-β, the large anharmonicity of the low-frequency vibrational modes decays more gradually than the other BEA materials, with the modes remaining mostly anharmonic until 400 cm^–1^, and the average mode-resolved anharmonicity is 0.58. Interestingly, deAl-β has greater anharmonic contributions at frequencies in the range of 700–900 cm^–1^ compared to other BEA materials, with σ_s_^A^ of 0.59, 0.59, and 0.57 for ω_s_ of 701.52, 725.38, and 866.63 cm^–1^, respectively, which correspond to Si–O vibrations of the silanol groups (Si–OH) on the defect site.^14,44^ Furthermore, Sn-β has two additional vibrational modes that appear in the band gap between 800 and 1000 cm^–1^, which is associated with the vibrational modes of Sn–O bonds.^14,45,46^ These modes of Sn–O bonds have moderate anharmonic contributions of 0.36 and 0.27. Overall, the mode-resolved analysis demonstrates that anharmonic contributions differ drastically for modes in the vibrational spectrum. Intuitively, the low-frequency acoustic phonon modes, which correspond to soft vibrations of the BEA framework, are the most anharmonic and are not captured in the harmonic picture at all. More interestingly, we also observe local changes in anharmonicity that can be attributed to changes in chemical reactivity, as discussed below.

### 3.3 Element-Resolved Anharmonicity

**Figure 6:**
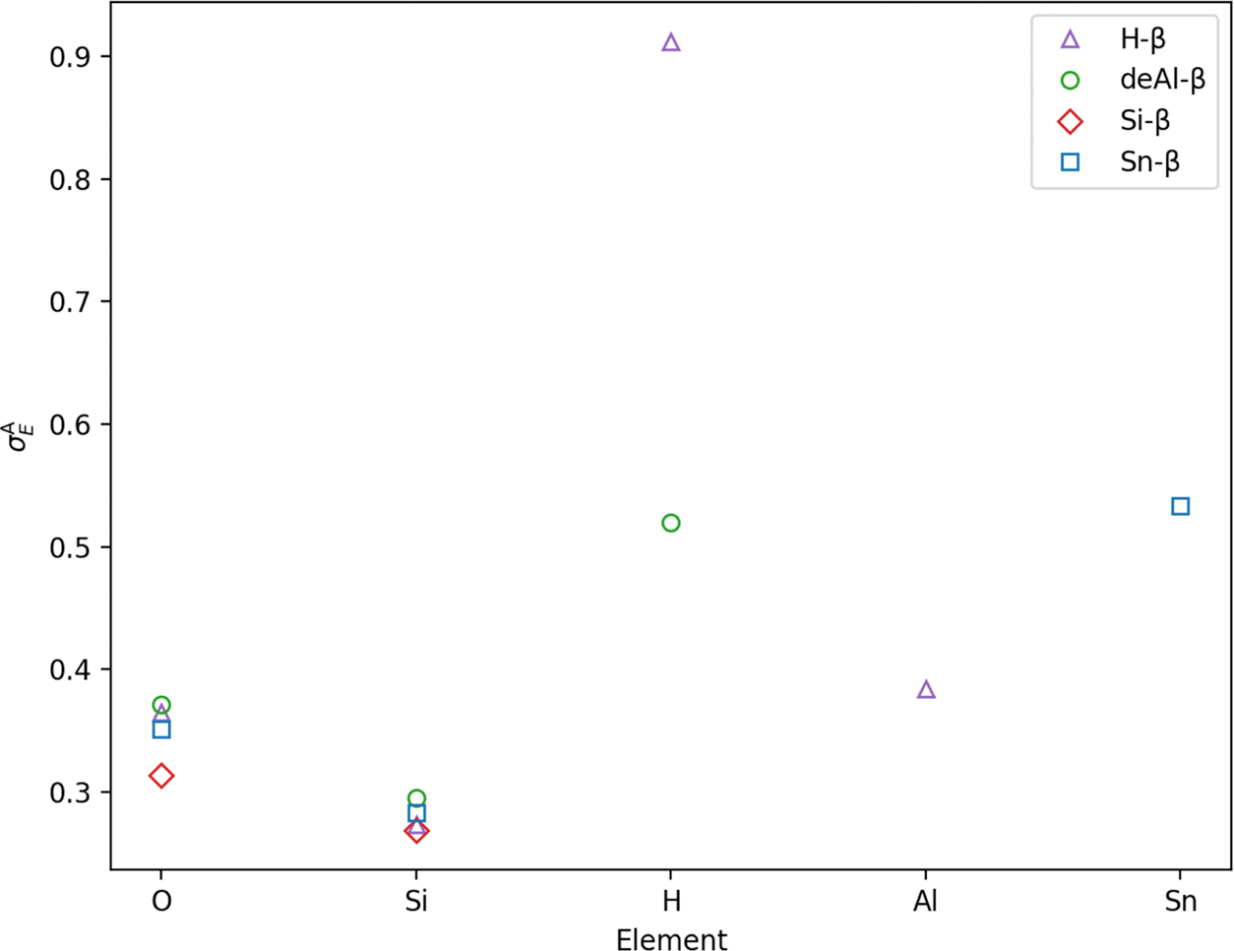
Element-resolved anharmonicity, σ_E_^A^, as appropriate for the same systems with the key provided in the top right corner. For all BEA materials, harmonic force constants were obtained through regression methods, and the PES was evaluated using harmonic sampling at 300 K.

Element-resolved anharmonicity, σ_E_^A^, which evaluates anharmonic contributions per chemical element, can also be obtained for each system (Figure 6). The anharmonic properties displayed in BEA materials can be driven by certain elements; for example, the anharmonic contribution of oxygen is broadly similar across all BEA materials, although oxygen has a slightly lower anharmonic contribution for Si-β of 0.31. For silicon, the anharmonic contribution is smaller than other elements; Si-β has the lowest anharmonic contributions from silicon, which explains the relatively low overall anharmonicity of Si-β when combined with the low anharmonic contributions from oxygen. Incorporation of heteroatoms into the BEA framework results in varying anharmonic contributions, with tin and aluminum having σ_E_^A^ of 0.53 and 0.38, respectively, *i.e.*, anharmonic contributions are one-third of the vibration for aluminum and greater than half of the vibration for tin. For hydrogen, anharmonic contributions are drastically different across BEA systems. In deAl-β, hydrogen atoms from the silanol (Si–OH) nest of the defect in the framework have anharmonic contributions of 0.52, while the hydrogen atom of the Brønsted acid site (Al–(OH)–Si) is almost entirely anharmonic for H-β, with σ_s_^E^ of 0.91. The significant variation in anharmonicity for hydrogen atoms when considering deAl-β and H-β suggests that anharmonicity within BEA materials is driven by local chemical environment as well as element type.

The high anharmonicity exhibited by H on the Bronsted acid site when performing element-resolved analysis, especially for H-β, contrasts with the empirical scaling factors applied to O–H vibrations,^47,48^ which typically have values >0.90,^49^*i.e.*, suggesting that O–H vibrations are predominantly harmonic. The high scaling factor values for O–H vibrations, particularly those obtained with GGA functionals, arise due to the error cancellation between the poor approximation of the energy landscape with semi-local DFT and the inadequacy of the harmonic approximation toward vibrations. For instance, an anharmonic mode described by a Morse potential would be “softer” than the harmonic approximation in one direction but “harder” in the other, as depicted in Figure 2, which would lead to cancellation in the scaling factor producing values close to unity; however, we believe the discrepancy observed here may also arise through application of different methods to sample energy landscapes. Pronounced anharmonicity could occur from the aiMD approach through the evaluation of atomic displacements that sample significant variation in the local environment, and hence interatomic interactions around the Brønsted acid site, which themselves are known to increase anharmonicity^50,51^ compared to the limited sampling along Cartesian axes that may be used to derive scaling factors for the harmonic approximation. Our results demonstrate that anharmonicity is dependent on chemical environment, and therefore another implication is the potential inadequacy of scaling factors derived for application to results *via* the harmonic approximation, if these scalars are then applied for the same bond types in different environments. For instance, in this work, σ_s_^E^ for hydroxyl group H atoms varies drastically depending on its chemical nature, *i.e.*, whether the O–H bond is a bridging group (0.91) or a part of a silanol group (0.52). The results suggest that to fully account for anharmonicity with scaling factors, exact considerations of chemical environment are necessary.

### 3.4 Atom-Resolved Anharmonicity

**Figure 7:**
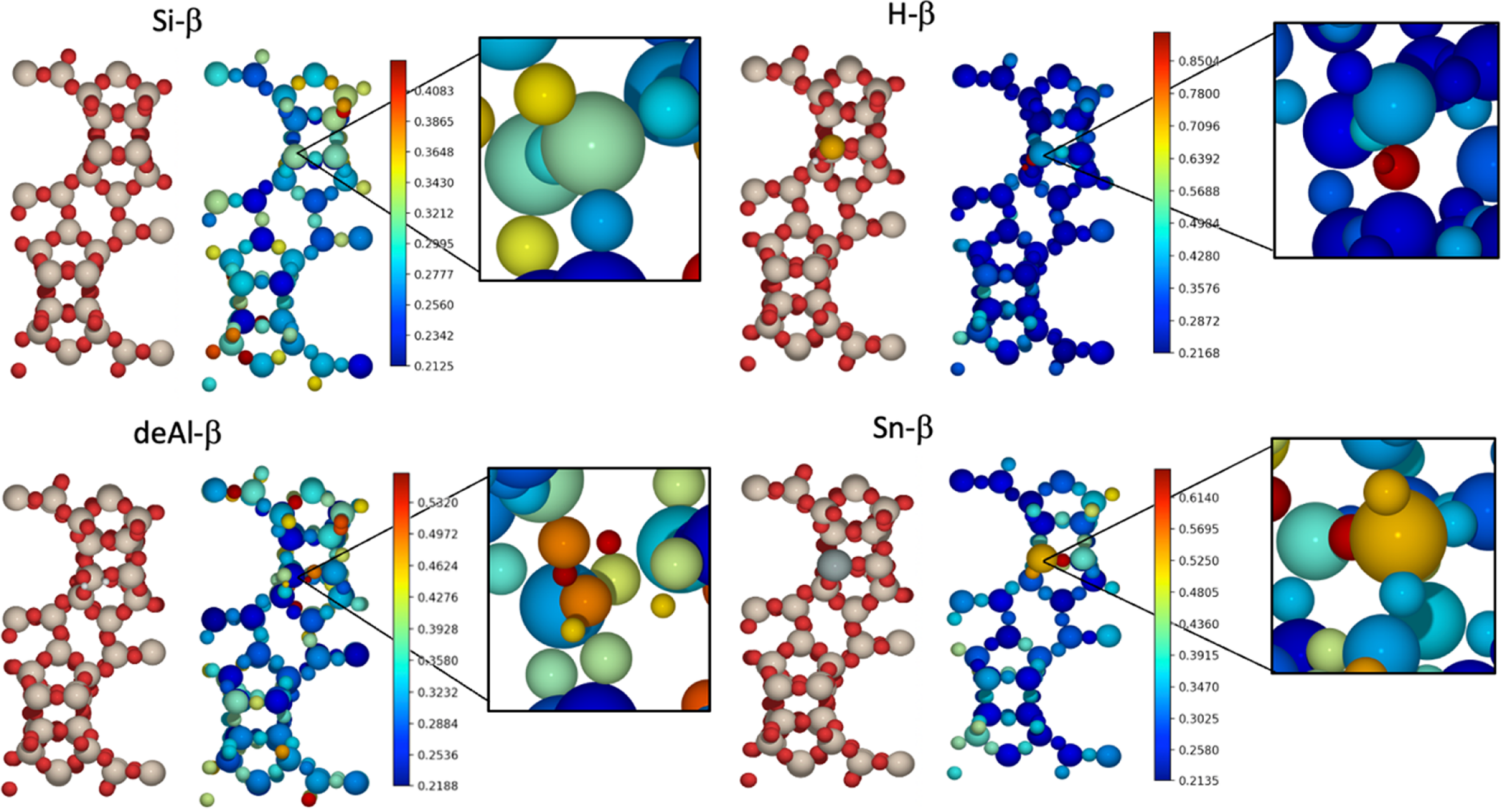
Color map (plotted to different scales) for anharmonic contribution per atom for the unit cells of Si-, H-, deAl-, and Sn-β (as labeled), with a view along the *b* and *c* unit cell vectors. Insets represent a magnified view of the T2 active site. For all BEA materials, harmonic force constants were obtained through regression methods, and the PES was evaluated using harmonic sampling at 300 K. The red, beige, white, black, orange, and gray atoms represent O, Si, H, C, Al, and Sn, respectively.

Atom-resolved anharmonicity can provide further insight into localized anharmonic contributions by spatially resolving onto the unit cell (Figure 7). In the case of Si-β, anharmonicity is predominately uniform throughout the framework, with clusters of greater anharmonic contributions (∼0.4) on the tetrahedral T6 site. In contrast, for H-, deAl-, and Sn-β, anharmonicity is concentrated around the substituted T2 site. For deAl-β, the largest anharmonic contributions occur at the silanol O–H groups, and the anharmonicity is similarly concentrated strongly on the site of Sn incorporation for Sn-β; and, in the case of H-β, the highest area of anharmonicity is on the Brønsted acid site, which supports the high element-resolved anharmonicity of H, as shown in Figure 6. Where the element-resolved anharmonicity (Figure 6) suggests that strong anharmonic contributions in different BEA materials arise due to the presence of different chemical species, the atom-resolved anharmonicity (Figure 7) demonstrates the localized nature of anharmonicity within the framework. The results from element- and atom-resolved anharmonicity may provide insight into the catalytic behavior of the BEA systems; for example, the strongly anharmonic nature of the hydrogen in H-β, as demonstrated in Figure 7, could be related to the Brønsted acidity of the material because anharmonicity can account for more facile bond dissociation. Similarly, for Sn-β, the anharmonic nature of the Sn substituent, which is a catalytic active site, could influence heat dissipation and, consequently, the feasibility for catalyzed reaction processes. From the difference in anharmonic behavior observed in H-β and Sn-β, it is possible to infer that anharmonicity may have greater significance for a zeolite with extra-framework Brønsted acid species when compared to Lewis acid intra-framework counterparts; further work is necessary to investigate the general applicability of this result to extra-framework Lewis acid species and variation in zeolite morphology.

### 3.5 Anharmonic Effects and IR Spectra

**Figure 8:**
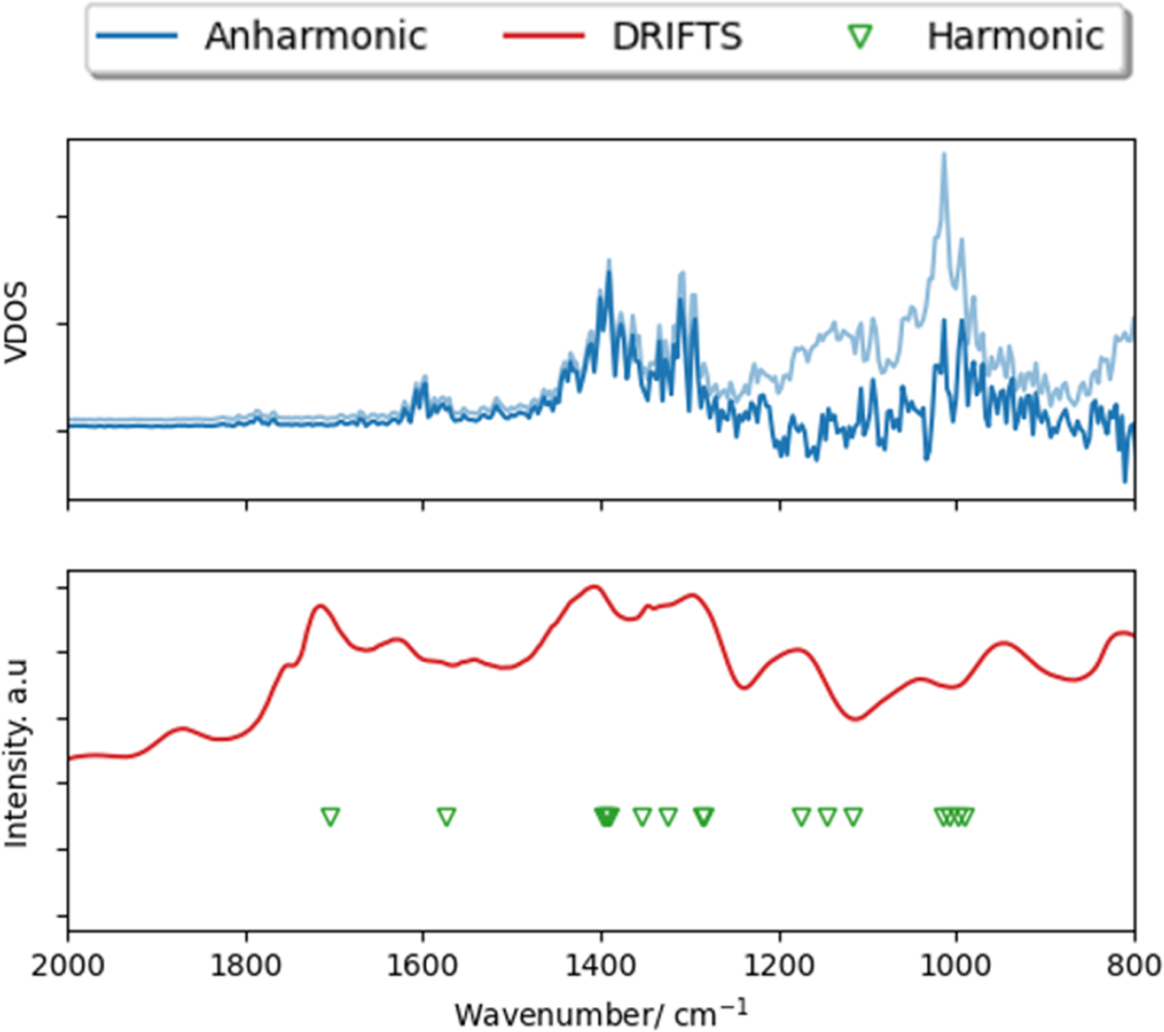
Top: Simulated VDOS (300 K) for framework-bound Sn(II) acetate (blue line) and for bound Sn(II) acetate/deAl-β (faded blue line). Bottom: Experimental DRIFTS spectra for Sn(II) acetate/deAl-β mixture (red line) and simulated IR frequencies using finite-difference method for framework-bound Sn(II) acetate (green triangles) replotted from ref [^14^].

Considering the significant anharmonicity displayed by BEA, it is necessary to consider the validity of harmonic models used for modeling vibrations in BEA and other zeolites. Studies in the literature have compared experimental IR spectra and VDOS from aiMD simulation to characterize the adsorption of alcohol in H-ZSM-5;^19^ however, there is little consideration for the role of anharmonic effects on the simulated vibrational modes. In order to understand the full impact of anharmonicity for the Sn-β system, we consider the DRIFTS spectrum of Sn(II) acetate adsorbed on deAl-β (Figure 8), which is a key step in SSI, as described in previous work.^14^ Subsequently, we compare the DRIFTS spectrum with calculated IR frequencies obtained using (i) the harmonic model (finite-difference method) and (ii) the aiMD approaches detailed here. Simulations using the harmonic model capture key features present in the DRIFTS spectrum, such as vibrational modes at ∼1360–1410 and 1720 cm^–1^ from carbonyl groups of Sn(II) acetate; however, vibrations containing anharmonic contributions, as obtained the vibrational density of states (VDOS) of aiMD data, are in far better agreement with DRIFTS spectra for smaller frequencies, ensuring a more complete profile of the vibrational modes is obtained. Considering the highly anharmonic behavior displayed by BEA systems and the better low-frequency representation of the vibrational spectrum obtained from an anharmonic treatment of vibrational modes, caution may be necessary when applying only harmonic models toward zeolites for modes <1500 cm^–1^.

Furthermore, the differences observed in the vibrational modes when applying the harmonic and anharmonic methodology have a direct impact on the change in entropy that can be calculated for reaction steps. Considering the adsorption of Sn(II) acetate on deAl-β, as introduced above, the change in entropy, Δ*S*, at 300 K is −223.26 J/K/mol when applying a harmonic model, compared to −307.44 J/K/mol when accounting for anharmonicity. The difference in Δ*S* suggests that including anharmonicity is important for accurate entropy and free energy calculations when studying these zeolite systems.

While the inclusion of anharmonic effects yields simulated spectra with improved agreement to experiment compared to the simple harmonic approaches, the quality of the representation of the PES remains unaddressed in this work and is important to consider in the future. The PES is defined by the choice of exchange–correlation functional in DFT, and improvements are necessary for further reconciliation between simulation and experiment. The impact of the exchange–correlation functional on computational vibrational modes has previously been reported in the literature. In particular, GGA functionals tend to overpredict H-bonding interactions, which leads to an overestimation of the change in OH frequencies upon adsorption.^16^ An overestimation in the change of frequencies will impact observables such as zero-point vibrational contributions, entropy, and, subsequently, adsorption enthalpy and free energy. The methods and systems considered in this work (aiMD, and unit cells of >192 atoms) were confined to the GGA level as higher-level methods were not computationally tractable; however, with the continued advent of more efficient computing paradigms, we hope that the quality of the representation of the PES can be addressed in the near future also.

## 4 Conclusions

In summary, anharmonic effects have been investigated for Si-β and BEA-structured intermediates present during the SSI process to form Sn-β, with the impacts identified *via* an anharmonicity score, σ^A^. Si-β has a greater anharmonicity (σ^A^ = 0.29) than dense silicate systems such as α-quartz (σ^A^ = 0.22), indicating that the porous nature of the framework and topology contribute to anharmonic behavior. For the aluminated BEA framework, the anharmonicity is increased, and the anharmonicity then decrease for SSI intermediates with a σ^A^ ranking of H-β (0.35) > deAl-β (0.34) > Sn-β (0.32); the result suggests that anharmonic behavior may influence Brønsted-acid zeolite catalysts more than their Lewis acid counterparts.

Vibrational mode-resolved analysis indicates that anharmonicity within BEA materials is strongest at low-frequency vibrational modes, which are almost entirely anharmonic because the porosity of the materials allows for flexible lattice distortions that are not included in simple harmonic models. Furthermore, elements can generate greater anharmonic contributions that can vary depending on the material. For silicon and oxygen, anharmonic contributions are relatively uniform and low (<0.4) across all BEA materials; however, heteroatoms such as aluminum and tin have varying anharmonic contributions, with tin having a σ_E_^A^ of 0.53 compared to 0.38 for aluminum, the latter being broadly similar to σ_E_^A^ displayed by silicon. Hydrogen displays drastically different anharmonic contributions depending on system type, with deAl-β having σ_E_^A^ for H of 0.53 and H-β being much greater at 0.91. The changes in anharmonicity for H demonstrate that anharmonic contributions are also dependent on local chemical environment.

The localized nature of the anharmonic effect is further supported by atom-resolved anharmonicity, which maps out anharmonic contributions across the BEA unit cell. For Si-β, anharmonicity is uniformly below 0.41; however, for H-, deAl-, and Sn-β, strong anharmonic contributions are concentrated on the T2 site, which is the site of dealumination and Sn incorporation in our models. The localized anharmonicity has possible consequences for catalytic activity at the active site.

In evaluating anharmonic effects within BEA systems, the factors that contribute toward anharmonicity have been identified. We highlight that the high degree of anharmonicity displayed by BEA raises questions about popular harmonic models used to simulate BEA and zeolites in general. Analysis of vibrational modes obtained from the simulated VDOS, which accounts for anharmonic effects, are in good agreement with DRIFTS data obtained from experiment, especially at low frequencies, when compared to vibrational modes obtained from a harmonic approximation. Given the significant anharmonic contributions measured in this work, further experimental analysis, e.g., *via* solid-state IR, would be a valuable next step. We also emphasize that improvements in the representation of the potential energy surface would be valuable to quantify the limitations of the semi-local exchange–correlation functional used in this work. The described results demonstrate the potential impact of anharmonicity on mechanistic aspects of applied zeolite catalysis, which will be investigated further.

## Supporting Information Available

Vibrational density of states as calculated with aiMD (20 K) and extracted via regression methods (hiPhive) for sodalite (blue line); convergence testing of the number of aiMD samples used to train hiPhive force constants; graphs of the average temperature, energy, and nuclear displacements during aiMD simulations; graphs of the mode-resolved anharmonicity of BEA using aiMD at 300 K; graphs of the average temperature, energy and nuclear displacement of Monte Carlo simulations for BEA materials; and similar graphs for Sn(II) acetate adsorbed in deAl-*β* (PDF)

## Notes

The accompanying supporting information also contains details of calculation methods. Information on the data underpinning the results presented here, including all structures, is available from the NOMAD repository (DOI: 10.17172/NOMAD/2023.06.30-1).
