# Supplementary material for: Evaluating the Role of Anharmonic Vibrations in Zeolite β Materials
Source: J Phys Chem C Nanomater Interfaces. 2023 Aug 7;127(32):16030–40. doi: 10.1021/acs.jpcc.3c02863 (PMC10440812; doi:10.1021/acs.jpcc.3c02863)
Supplement: Supplementary file 1 — jp3c02863_si_001.pdf [file jp3c02863_si_001.pdf]

Supporting Information

Evaluating the Role of Anharmonic Vibrations in Zeolite β Materials

Owain T. Beynon[1], Alun Owens[1], Christian Carbogno[2], Andrew J. Logsdail[1]**

1. Cardiff Catalysis Institute, Cardiff University, Park Place, Cardiff, CF10 3AT, Wales, UK

2. Fritz-Haber-Institut der Max-Planck-Gesellschaft, Faradayweg 4–6, D-14195 Berlin, Germany

*LogsdailA@cardiff.ac.uk, Christian.Carbogno@fhi-berlin.mpg.de

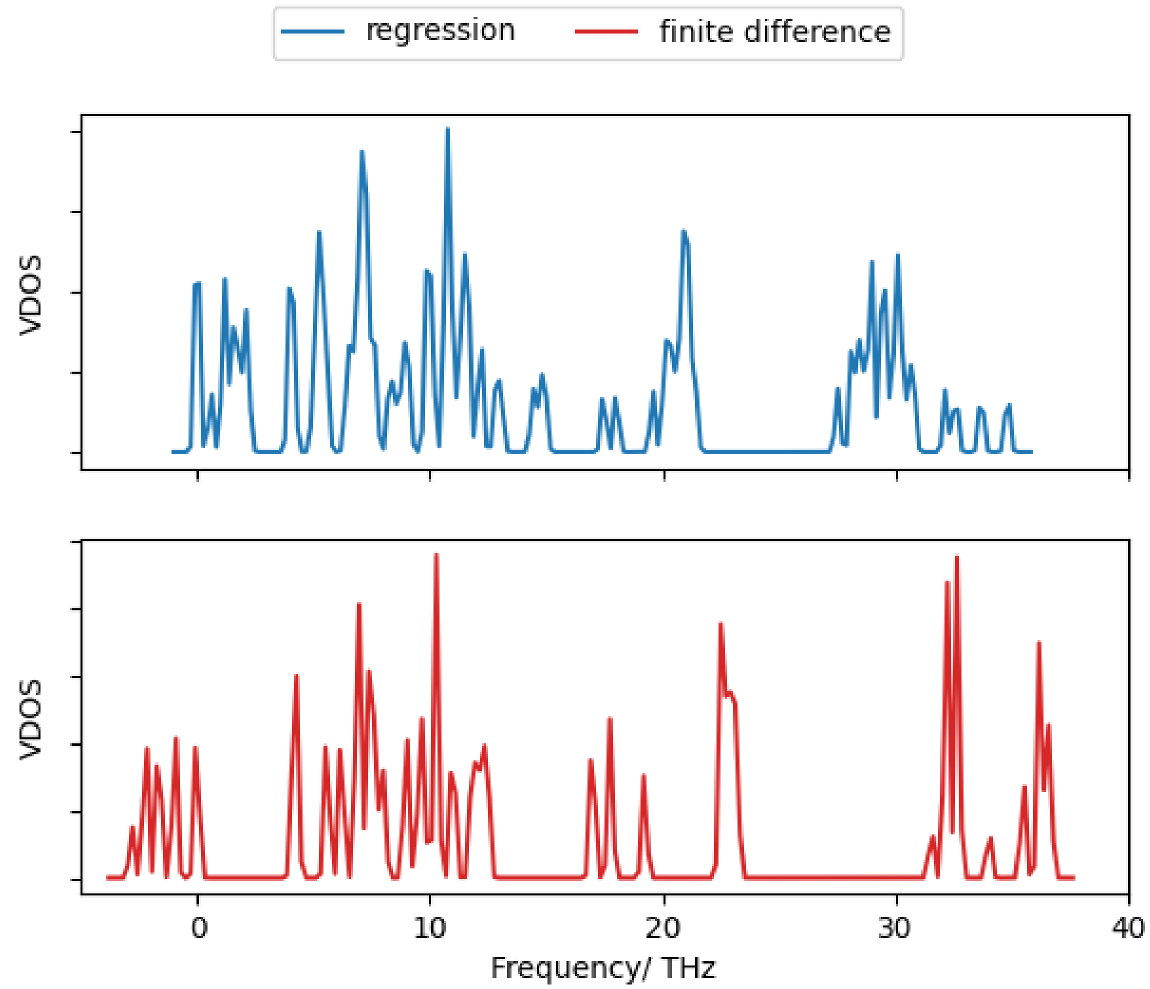

Figure S1. Top: vibrational density of states as calculated with aiMD (20 K) and extracted via regression methods (*hiPhive*) for sodalite (blue line). Bottom: vibrational density of states as calculated with *phonopy* (harmonic approximation/finite difference method with Γ-point sampling at 0 K) for sodalite (red line).

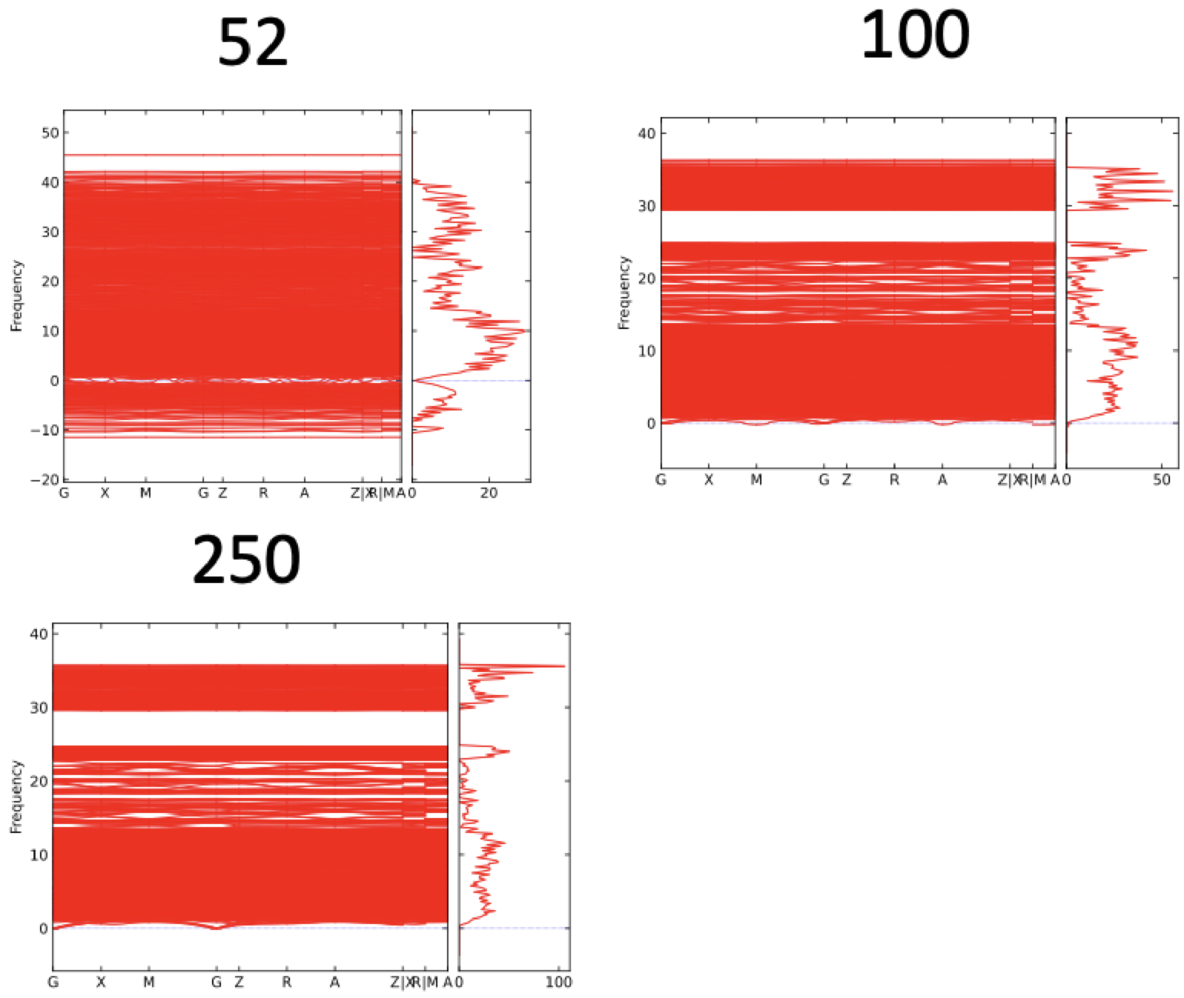

Figure S2. Convergence testing of the number of aiMD samples (as labelled) used to train *hiPhive* force constants. The phonon density of states (DOS) is plotted on the right of each figure.

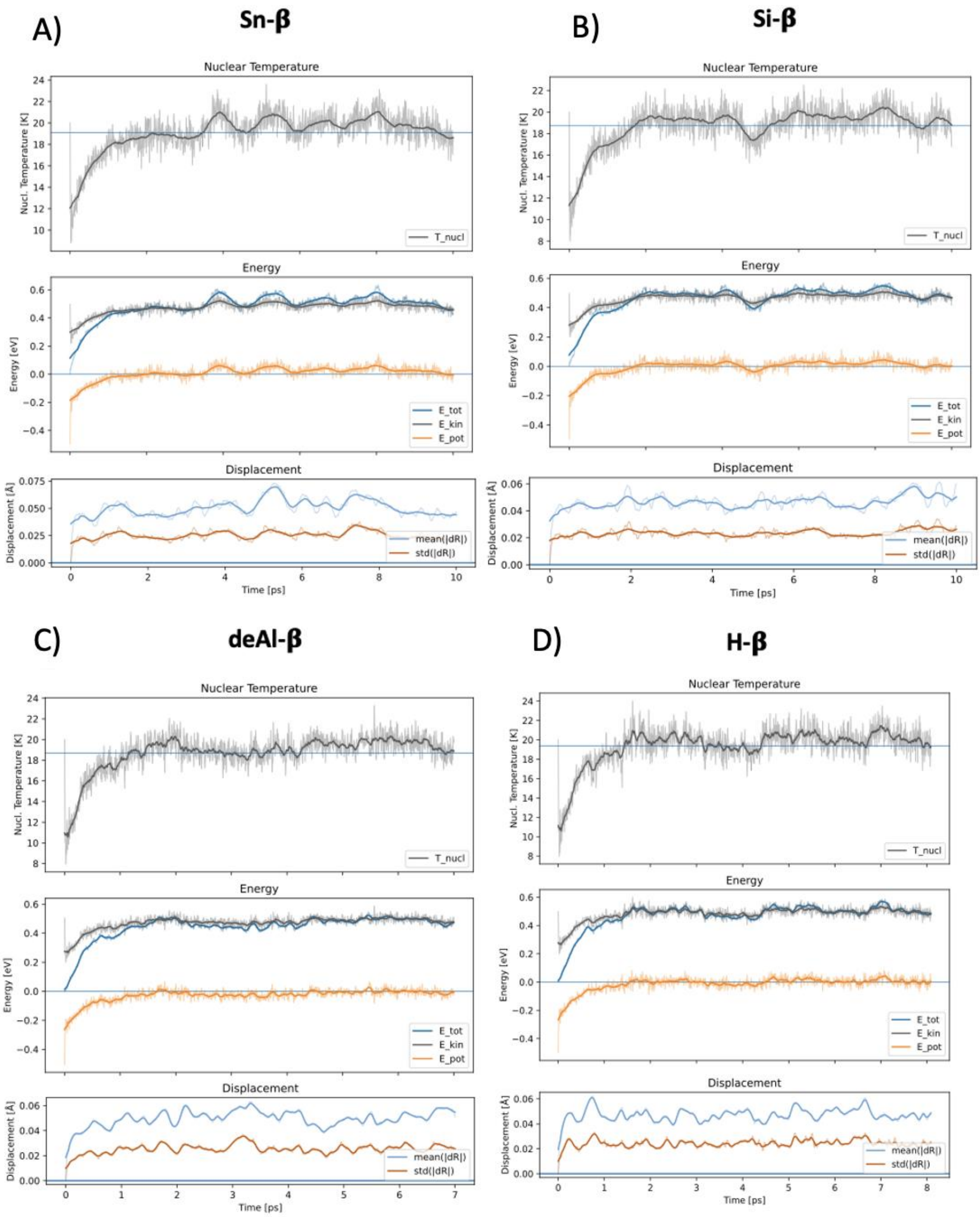

Figure S3. The average temperature, energy, and nuclear displacements during aiMD simulations for a) Sn-β b) Si-β c) deAl-β, d) H-β.

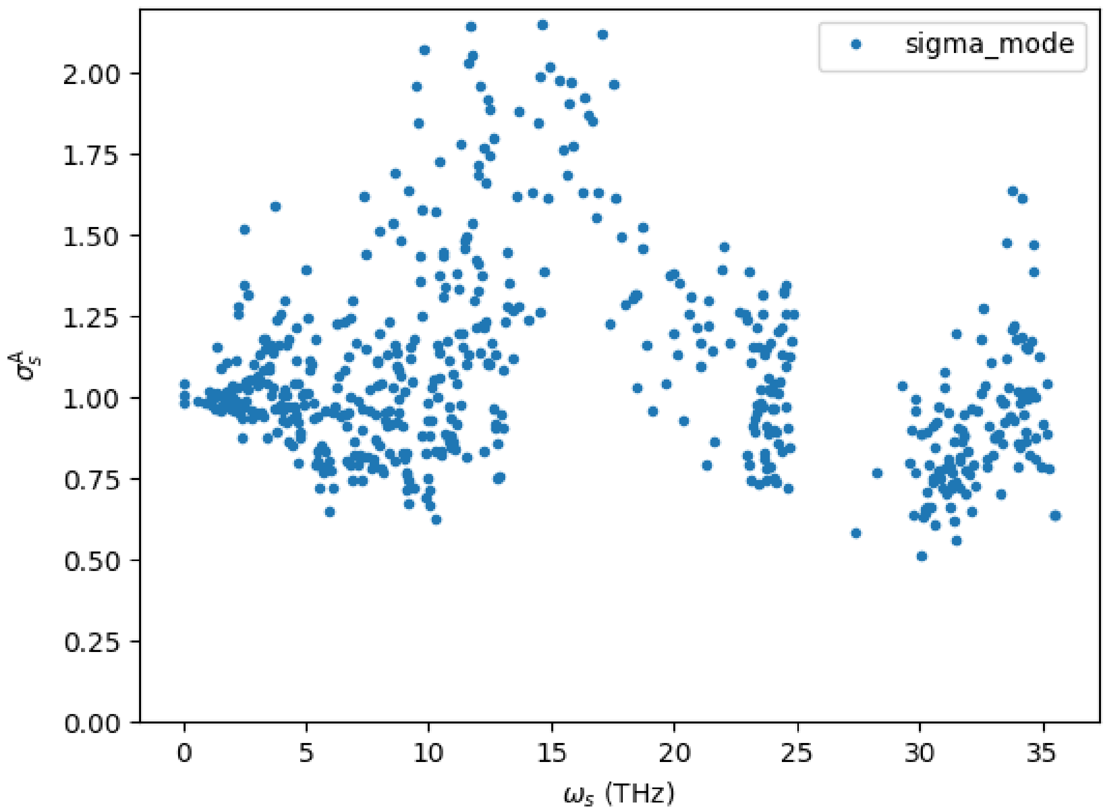

Figure S4. The mode-resolved anharmonicity of Si-β using aiMD at 300 K.

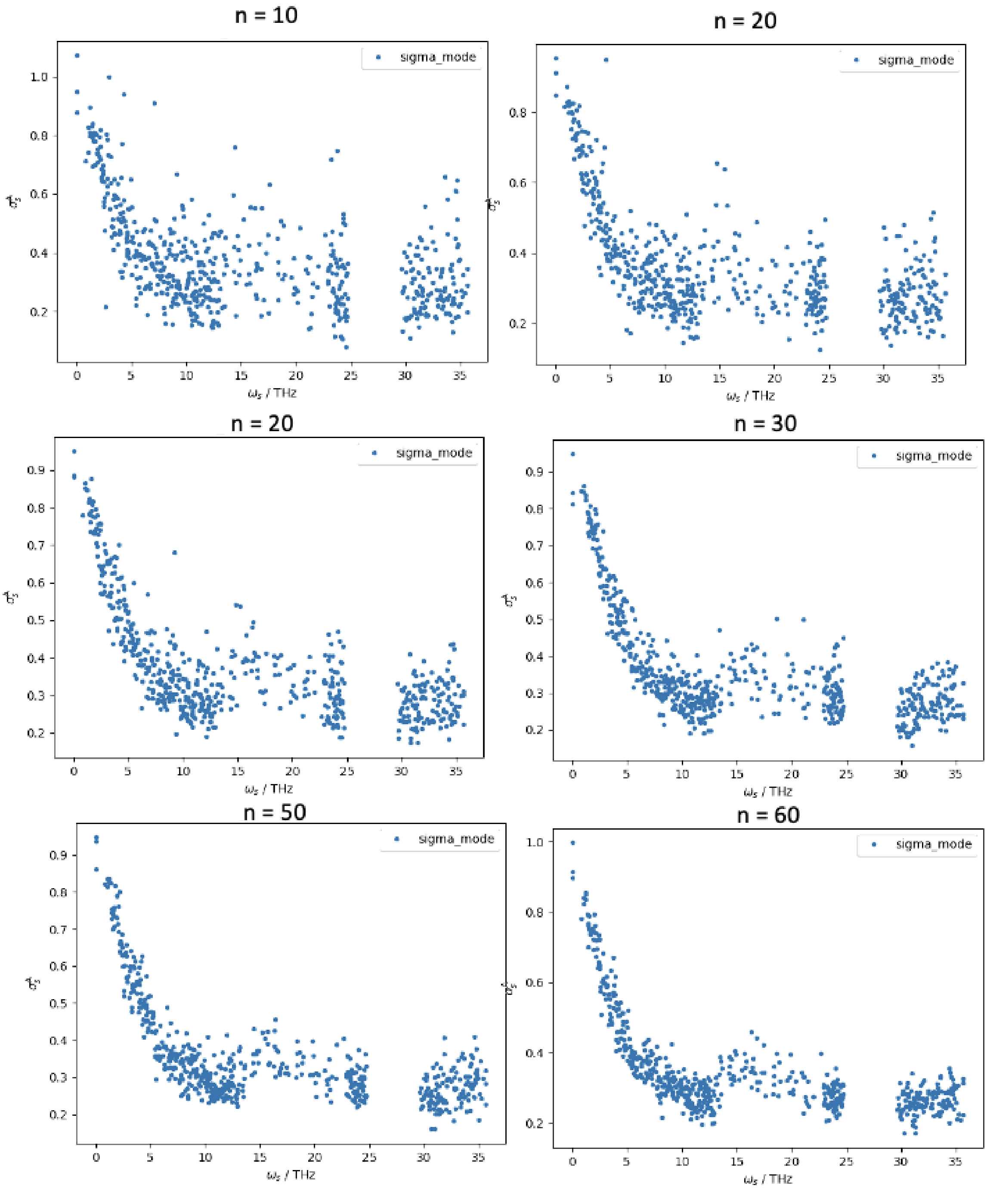

Figure S5. The mode-resolved anharmonicity for Si-β applying harmonic sampling, where n represents the number of Monte Carlo samples used.

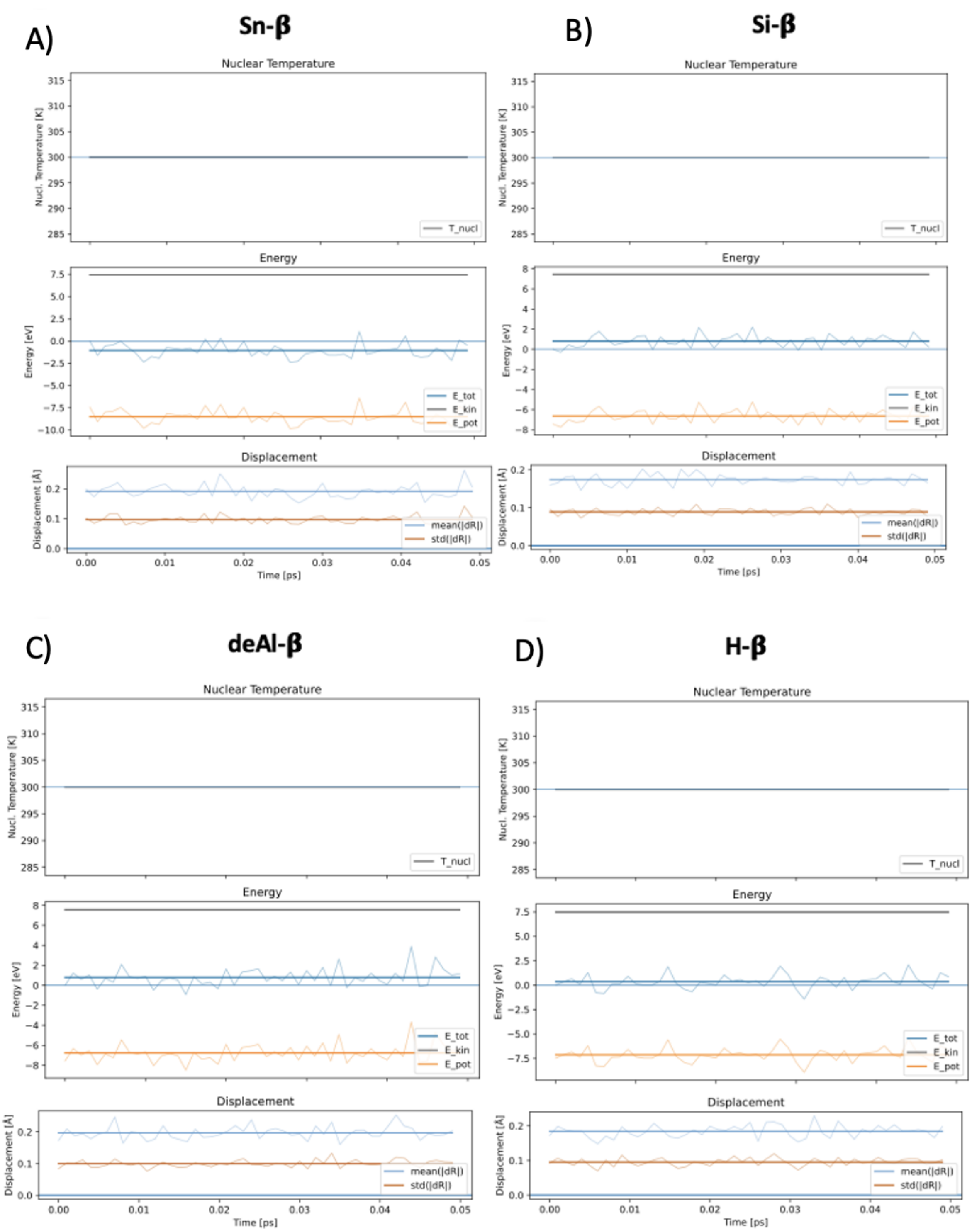

Figure S6. The average temperature, energy, and nuclear displacement of Monte Carlo simulations for a) Sn-β b) Si-β c) deAl-β, d) H-β.

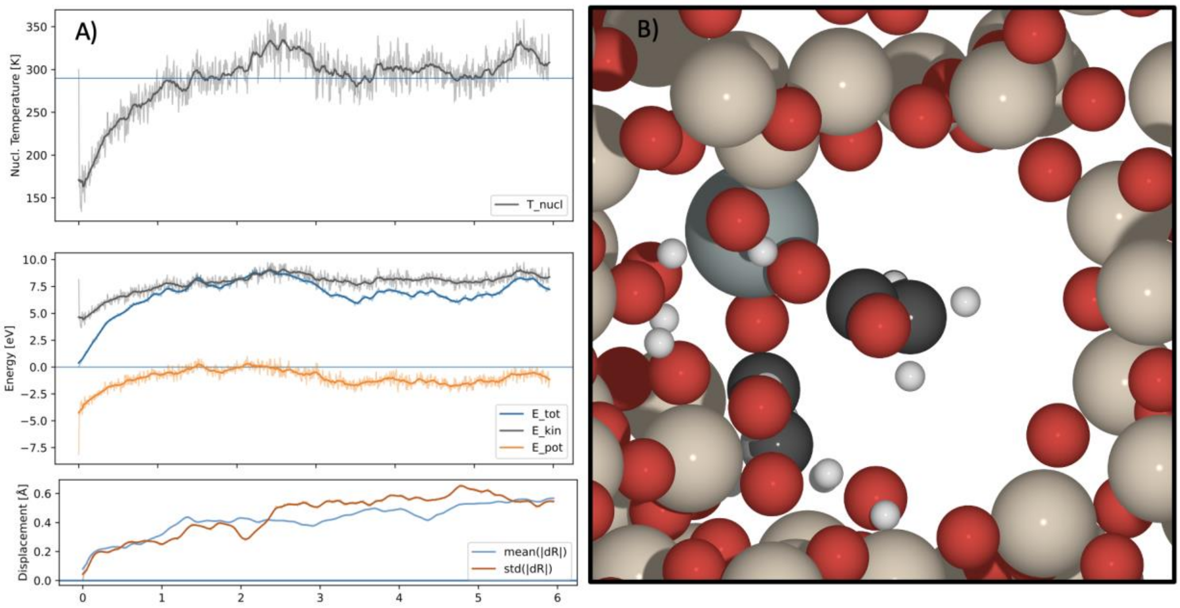

Figure S7. A) The average temperature, energy, and nuclear displacement of aiMD simulations for monodentate Sn(II) acetate in deAl-β; B) The model of monodentate Sn(II) acetate in deAl-β, where beige, red, grey, dark grey, and white atoms represent Si, O, C, Sn, and H respectively.

Computational Methods for Sodalite

DFT calculations were performed with the *Fritz Haber Institute Ab Initio Molecular Simulation* (*FHI-aims*) software package,[1] which is an all electron, full potential code. The general gradient approximation (GGA) exchange-correlation functional (XC) of Perdew-Burke-Ernzerhof, reparametrized for solids (PBEsol) was used,[2] with dispersion interactions accounted for using the Tkatchenko-Scheffler method.[3] Calculations were performed using a 'light' basis set of the 2010 release, with self-consistent field (SCF) cycle convergence reached when the sum of eigenvalues and change in charge density were below 10^{-6} e/a_0^3 and 10^{-6} eV, respectively. Calculations were also performed spin-restricted and using the zeroth order regular approximations (ZORA) for relativistic treatments[4].

The SOD unit cell obtained from the database of international zeolite association (IZA).[5] Models were built and manipulated using the Atomic Simulation Environment (*ASE*) and *FHI-vibes* Python libraries.[6,7] Electronic structure calculations were performed with a converged Monkhorst-Pack **k**-point sampling grid of 6x6x6. Full unit cell optimisations were performed on all structures using the Broyden-Fletcher-Goldfarb-Shanno (BFGS) algorithm with convergence reached when the forces on all atoms are less than a strict criterion of 0.001 eV $Å^{-1}$.[8–11] Anharmonic contributions were calculated using *FHI-vibes*. *Ab initio* Molecular Dynamics (aiMD) simulations were performed with *FHI-vibes*, *FHI-aims*, and *ASE*, using Langevin dynamics. Harmonic force constants were directly obtained using the *phonopy* package, with Γ-point sampling, and were obtained from aiMD simulations using the *hiPhive* Python package.[12,13]

References

(1) Blum, V.; Gehrke, R.; Hanke, F.; Havu, P.; Havu, V.; Ren, X.; Reuter, K.; Scheffler, M. Ab Initio Molecular Simulations with Numeric Atom-Centered Orbitals. *Computer Physics Communications* **2009**, *180* (11), 2175–2196.

(2) Perdew, J. P.; Ruzsinszky, A.; Csonka, G. I.; Vydrov, O. A.; Scuseria, G. E.; Constantin, L. A.; Zhou, X.; Burke, K. Restoring the Density-Gradient Expansion for Exchange in Solids and Surfaces. *Phys. Rev. Lett.* **2008**, *100* (13), 136406.

(3) Tkatchenko, A.; Scheffler, M. Accurate Molecular Van Der Waals Interactions from Ground-State Electron Density and Free-Atom Reference Data. *Phys. Rev. Lett.* **2009**, *102* (7), 073005.

(4) van Lenthe, E.; Baerends, E. J.; Snijders, J. G. Relativistic Total Energy Using Regular Approximations. *The Journal of Chemical Physics* **1994**, *101* (11), 9783–9792.

(5) Baerlocher, C.; McCusker, L. *Database of Zeolite Structures.* http://www.iza-structure.org/databases/

(6) Hjorth Larsen, A.; Jørgen Mortensen, J.; Blomqvist, J.; Castelli, I. E.; Christensen, R.; Dułak, M.; Friis, J.; Groves, M. N.; Hammer, B.; Hargus, C.; Hermes, E. D.; Jennings, P. C.; Bjerre Jensen, P.; Kermode, J.; Kitchin, J. R.; Leonhard Kolsbjerg, E.; Kubal, J.; Kaasbjerg, K.; Lysgaard, S.; Bergmann Maronsson, J.; Maxson, T.; Olsen, T.; Pastewka, L.; Peterson, A.; Rostgaard, C.; Schiøtz, J.; Schütt, O.; Strange, M.; Thygesen, K. S.; Vegge, T.; Vilhelmsen, L.; Walter, M.; Zeng, Z.; Jacobsen, K. W. The Atomic Simulation Environment—a Python Library for Working with Atoms. *J. Phys.: Condens. Matter* **2017**, *29* (27), 273002.

(7) Knoop, F.; Purcell, T.; Scheffler, M.; Carbogno, C. FHI-Vibes: Ab Initio Vibrational Simulations. *JOSS* **2020**, *5* (56), 2671.

(8) Broyden, C. G. The Convergence of a Class of Double-Rank Minimization Algorithms 1. General Considerations. *IMA J Appl Math* **1970**, *6* (1), 76–90.

(9) Fletcher, R. A New Approach to Variable Metric Algorithms. *The Computer Journal* **1970**, *13* (3), 317–322.

(10) Goldfarb, D. A Family of Variable-Metric Methods Derived by Variational Means. *Math. Comp.* **1970**, *24* (109), 23–23.

(11) Shanno, D. F. Conditioning of Quasi-Newton Methods for Function Minimization. *Math. Comp.* **1970**, *24* (111), 647–647.

(12) Eriksson, F.; Fransson, E.; Erhart, P. The Hiphive Package for the Extraction of High-Order Force Constants by Machine Learning. *Adv. Theory Simul.* **2019**, *2* (5), 1800184.

(13) Togo, A.; Tanaka, I. First Principles Phonon Calculations in Materials Science. *Scripta Materialia* **2015**, *108*, 1–5.
